# An evaluation of mathematical models for the outbreak of COVID-19

**DOI:** 10.1093/pcmedi/pbaa016

**Published:** 2020-05-22

**Authors:** Ning Wang, Yuting Fu, Hu Zhang, Huipeng Shi

**Affiliations:** Mathematical Institute, University of Oxford, Radcliffe Observatory Quarter, Woodstock Road, Oxford OX2 6GG, UK; Mathematical Institute, University of Oxford, Radcliffe Observatory Quarter, Woodstock Road, Oxford OX2 6GG, UK; Department of Gastroenterology & Center for Inflammatory Bowel Disease, West China Hospital, Sichuan University, Chengdu 610041, China; Department of Orthopaedics, Shanghai 6th People’s Hospital, Shanghai Jiaotong University, Shanghai 201306, China

**Keywords:** COVID-19, 2019-nCoV, SARS-CoV-2, novel coronavirus, epidemiological modelling, SIR model, SEIR model, case fatality ratio, basic reproduction numbers, asymptomatic infections, herd immunity, intervention measures

## Abstract

Mathematical modelling performs a vital part in estimating and controlling the recent outbreak of coronavirus disease 2019 (COVID-19). In this epidemic, most countries impose severe intervention measures to contain the spread of COVID-19. The policymakers are forced to make difficult decisions to leverage between health and economic development. How and when to make clinical and public health decisions in an epidemic situation is a challenging question. The most appropriate solution is based on scientific evidence, which is mainly dependent on data and models. So one of the most critical problems during this crisis is whether we can develop reliable epidemiological models to forecast the evolution of the virus and estimate the effectiveness of various intervention measures and their impacts on the economy. There are numerous types of mathematical model for epidemiological diseases. In this paper, we present some critical reviews on mathematical models for the outbreak of COVID-19. Some elementary models are presented as an initial formulation for an epidemic. We give some basic concepts, notations, and foundation for epidemiological modelling. More related works are also introduced and evaluated by considering epidemiological features such as disease tendency, latent effects, susceptibility, basic reproduction numbers, asymptomatic infections, herd immunity, and impact of the interventions.

## Introduction

The coronavirus disease 2019 (COVID-19), declared as a pandemic by the World Health Organization at the end of January 2020, is considered to be the most devastating infectious disease outbreak ever since the 1918 influenza pandemic. The first case of COVID-19 was reported on 31 December 2019 in Wuhan China, which was initially identified as the epicentre of the virus.^1^ Given it is apparently able to generate rapid and substantial human-to-human transmissions, COVID-19 has largely been spreading in vast geographic regions including more than 200 countries and severely hit countries such as Iran, Italy, Spain, France, Germany, UK, and US, etc. As of 19 April 2020, about 2 394 278 confirmed cases including more than 164 937 deaths had been reported worldwide according to the COVID-19 map by the Johns Hopkins University,^2^ which reveals that the epicentre is gradually moving to Europe and America. Even though serious measures were being undertaken by the vast majority of countries to contain the outbreak of COVID-19, it is still spreading globally in an overwhelming manner.^3^

**Figure 1.**
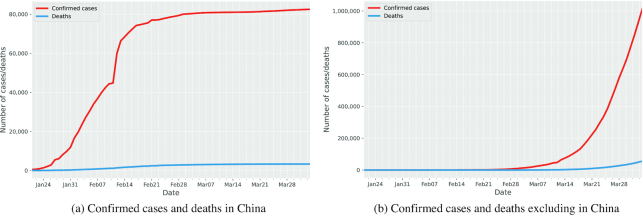
Global confirmed cases and deaths for COVID-19.

**Figure 2.**
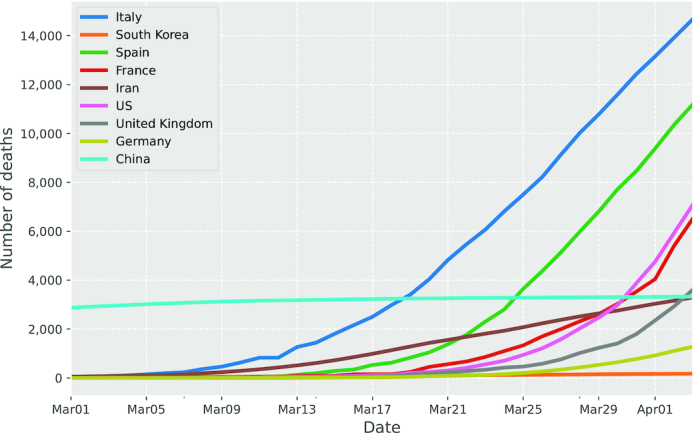
Global COVID-19 deaths since March 2020.

In general, an epidemic follows a similar tendency, which can be modelled mathematically. Initially, the number of infected cases progressively increased, which usually exhibits exponential behaviour. On reaching the peak, it then turns over and gradually decreases. Ultimately, the outbreak fades out to zero, which implies the end of the epidemic. From the data in Ref. 4. we can observe from Fig. 1a that the infected cases in China started to grow in January 2020 and there was a sudden increase within a single day in February 2020 due to the change of diagnostic methods. Then the curve of China began to be saturated after early March. We can see from Fig. 2 that China and Republic of Korea seem to have been in the decreasing phase in April thanks to some serious preventive measures including lockdown of cities, closure of schools, shops and public transport, tracing and isolating infected individuals, and so on. These prevention measures lead to a sudden decline in transmission rate and turn out to be an effective strategy in containing the virus. For other countries, such as Italy, Spain, France, Germany, UK, and the US, we can observe from Fig. 1b that the number of cases is still in the rapid increase phase and some curves are still yet to reach the peak.

As an utterly novel disease, COVID-19 has a considerable effect on public health and the global economy. Mathematical modelling plays a vital part in predicting and controlling the ongoing COVID-19 pandemic. One of the most significant problems during the pandemic is whether we can develop mathematical models to predict the evolution of the epidemic, and estimate the effectiveness of various intervention measures and their impact on the economy. The models widely affect policy and diagnostic decision-making. There are typically three categories of mathematical model for epidemiology, namely empirical models, including machine-learning, statistical, and dynamical methods.^5^ At this early stage, it is feasible to pay more attention to the dynamical model due to the insufficient data on this pandemic. Epidemiological models incorporate deterministic or stochastic methodologies based on different sub-group populations. The most notably deterministic models include susceptible-infectious-recovered (SIR), susceptible-infectious-susceptible (SIS), and susceptible-exposed-infectious-susceptible (SEIS) models.^6,7^ In this paper, we highlight some essential mathematical models for epidemiological analysis and review recent papers concentrating on the trend and the severity of COVID-19, the impact of asymptomatic infection, and the effect of intervention measures.

## Mathematical models

Mathematical models are efficient tools to understand the ongoing trends for COVID-19. The models are essential to make a therapeutic choice when surge capacity has been exceeded or without ready access to laboratory testing.^8^ The models are crucial for the policymaker to acquire medical supplies, allocate human resources and hospital beds, and ensure the sustainability of the health system throughout the peak and duration of the epidemic.^8^ Researchers around the world have performed numerous mathematical modelling and numerical analysis on COVID-19 since its outbreak. In the following section, we review these proposed methods and present the updated results with focus on the following topics: fatality ratio, disease tendency, basic reproduction numbers, asymptomatic infective, herd immunity, and the effects of intervention measures.

### Case fatality ratio

Severity is one of the most concerning factors in the outbreak of a pandemic. The fatality ratio is a crucial measurement to describe the severity of the transmittable disease. It is a challenging task to predict the fatality rate as it changes over time and can be measured in many different ways during an epidemic. The case fatality ratio (CFR) is a standard measurement that estimates the proportion of deaths from the disease to the total number cases diagnosed with the disease. A common variant of CFR is the delayed CFR defined as follows.^9^

\begin{eqnarray*} \mathrm{CFR}_{\mathrm{d}}\left(t, \tau _{\mathrm{res}}\right)=\frac{D (t)}{N\left(t-\tau _{\mathrm{res}}\right)} \end{eqnarray*}

where *D*(*t*) represents the number of deaths at time *t; N*(*t* − τ_res_) specifies the number of diagnosed cases in the period (*t* − *τ*_res_); and *τ*_res_ denotes a corresponding time lag indicating the duration from the day when the first symptoms occurred to the day of outcome (recovery or death).

Wu and McGoogan estimate CFR_d_(*t*, 0) of COVID-19 in China to be 2.3% on 11 February 2020, which assumes that *τ*_res_ equals zero and thereby underestimates the true CFR.^10^ With the help of more public information, Wu *et al*. calculate that the overall symptomatic CFR (the probability of death after developing symptoms) of COVID-19 in Wuhan is 1.4% (0.9%–2.1%),^8^ which is noticeably less than both the naive CFR (2169/48 557 = 4.5%) and the approximation of fatality rate (2169/(2169 + 17 572) = 11%) as of 29 February 2020.

Böttcher *et al*. compare the individual- and population-based CFR. The former one is the probability that an individual who has been infected for time *τ*_1_ died by time *t*; the latter is the ratio of the number of death cases over the number of cases with the outcome (death/recovery).^11^ They point out that only when the recovery rate and death rate are constant, the population-based CFR becomes irrelevant from disease transmission at the population level and coincides with the individual-based CFR.^11^ The population-based CFR is generally not as useful as individual-based CFR because: (i) the infection time distributions that are important on an individual level might not match those in population level; and (ii) the population-level mortality ratios tend to be time-dependent and only in the stable state as soon as the outbreak ends.

Dorigatti *et al*. estimate both individual- and population-based CFR of COVID-19.^12^ They first evaluate the intervals between onset of symptoms and outcome. Here, we denote *f_OD_*(.) as the probability density function (PDF) associated with the time from symptoms occur to death, which follows a gamma distribution. Then the PDF that a death is observed at time *t_d_* with presumed onset *τ* days ago is defined as follows.

\begin{eqnarray*} g_{O D}\left(\tau | t_{d}\right)=\frac{f_{O D} (\tau ) o\left(t_{d}-\tau \right)}{\int _{0}^{\infty } f_{O D}\left(\tau ^{\prime }\right) o\left(t_{d}-\tau ^{\prime }\right) d \tau ^{\prime }} \end{eqnarray*}

where *o*(*t*) is the observed number of onsets that occurred at time *t* assuming exponential growth. Similarly, the posterior onset-to-recovery distribution *g_OD_*(.) can be fitted. Using data from China and assuming uniform prior distribution, the onset-to-recovery is 22.2 days (18–83, 95% CI) and the onset-to-death is 22.3 days (18–82, 95% CI). Based on the posterior of *f_OD_*(.) the overall probability of all observed deaths, recoveries, and cases residing in hospital can be formulated from the individual case data. The posterior distribution of CFR can be obtained by applying Bayes' rule. The CFR can also be estimated from the aggregated population cases data (daily reports of cases). Denote *D*(*t*) and *C*(*t*) as the incidence of deaths and onsets, the anticipated number of deaths at time *t* is given by

\begin{eqnarray*} D (t)\,=\,c \int_{0}^{\infty } C (t-\tau ) f_{O D} (\tau ) d \tau \end{eqnarray*}

where *c* is the CFR that can be estimated with Bayes rule. They estimate the CFR of Hubei to be 18% (11%–81%, 95% CI) on 5 February 2020 and the CFR outside mainland China to be 5.1% (1.1%–38%, 95% CI) using death and recovery outcomes. Assume that infected individuals test positive from *l* days prior to onset of symptoms to *n* − *l* days later. An infection prevalence at time *t, y*(*t*) is generally approximated as

\begin{eqnarray*} y (t,=\,\int_{-l}^{n-l} C (t-\tau) d \tau / N \end{eqnarray*}

where *N* indicates the population of the region. Based on data of repatriation flights, they estimate the CFR among all infections to be 0.9% (0.5%–4%, 95% CI) supposing that clinical symptoms are observable after 14 days, and the CFR is 0.8% (0.4%–3.1%, 95% CI) providing disease is noticeable after 7 days.

### Susceptible-Infected-Removed (SIR) model

**Figure 3.**
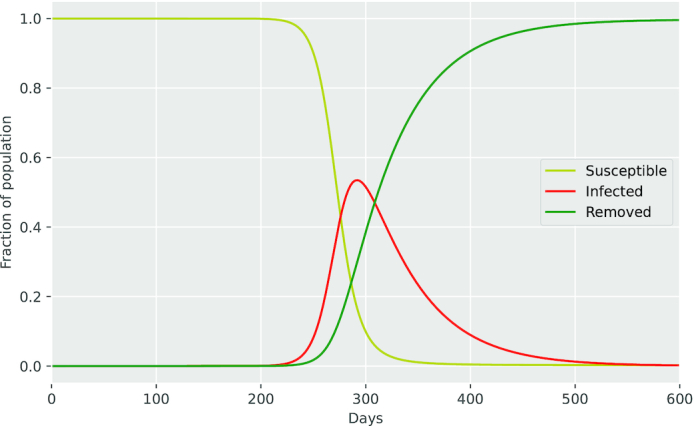
SIR model.

The purpose of disease control is to build a predictive model in an effort to better understand the key factors that influence COVID-19 transmission. The SIR model is one of the fundamental epidemiological models that illustrates the dynamics of an infective epidemic given that large population has already been susceptible, infected, and recovered. Assuming that the virus is generally contracted only once, an infected individual either passes away or recovers. As the disease spreads, the susceptibles are likely to get infected, and the infected individuals tend to be removed (either die or recover). It presumes that the total number of the population is a constant number of *N* and the total infected population eventually goes into the removed category. The model divides a homogeneous and isolated population into the following three categories:

- Susceptible (*S*). These have not contracted the virus but might be infected as a result of transmission from an infected individual. As demonstrated in Fig. 3, the *S* initially reveals a rapid decrease, and it eventually fades out and turns into zero.
- Infected (*I*). These have already contracted the disease. It is indicated in Fig. 3 that the *I* at first exhibits a sluggish increase. As time passes, it shifts into a sharp growth prior to the maximum value.
- Removed (*R*). The virus likely leads to one of two directions: either the person recovers or dies, which are treated equally in this model. As shown in Fig. 3, the *R* consequently illustrates a saturation as soon as the peak value appears to have been surpassed.

We denote by *S*(*t*), *I*(*t*), and *R*(*t*) the populations of these classes at time *t* and assume *S*(*t*) + *I*(*t*) + *R*(*t*) = *N* for all *t*.

The model is described by the following equations where *β* is the contact rate and *μ* is the removal rate.

\begin{eqnarray*} \frac{dS}{dt} &=& -\beta SI \\ \frac{dI}{dt} &=& \beta SI -\mu I \\ \frac{dR}{dt} &=& \mu I \end{eqnarray*}

The SIR model considered here is the simplest prototype. The spread of an infectious virus involves not only disease-related factors such as the infectious agent, mode of transmission, latent period, infectious period, susceptibility, and resistance, but also social, cultural, demographic, economic, and geographic factors.^7^ As the epidemic consistently spreads across the world, the SIR model presents extremely limited dynamics to capture the complexity of the outbreak of COVID-19. For that reason, various refined models are proposed to accommodate more data sources and enrich with additional real-time data in order to lead the decision-making for public health.

The SIR model usually fixes specific parameters such as the contact rate and the removal rate. Wu,*et al*. extend the model by updating the transmission rate and recovery rate based on historical data.^8^ The authors estimate the clinical severity of COVID-19 using the symptomatic CFR (sCFR), which specifies an infectious individual who is detected with the specific clinical symptoms. They simulate the epidemic based on a modified SIR model considering the travel information by combining the eight data sources, including confirmed cases, death cases, age of confirmation and death cases, the time between onset and death, traveller, and so on. They estimate the parameters for different age groups, including sCFR by Markov Chain Monte Carlo (MCMC) methods with Gibbs sampling and non-informative flat priors.^8^ The estimation of sCFR is about 1.4% (0.9%–2.1%), which is not sensitive to infection-symptomatic probability *P_sym_* (the probability of the infections that develop symptoms). As one of very early publication on COVID-19, the paper may marginally underestimate the epidemic as they only incorporate the first 425 cases in Wuhan with the assumption that travellers are well enough to travel, which not includes those who are already in a severe condition and possibly in hospital.

Chen *et al*. propose a time-dependent SIR model to estimate the quantity of confirmed cases of COVID-19.^13^ They convert the SIR model into a discrete-time model using ridge regression to track two time-series data; namely, the transmission rate and the recover rate at time *t*. The proposed model conforms to various disease control measures, most notably the lockdown of cities. The model presents that the daily prediction errors for the confirmed cases in China are lower than 3% apart from the day when there was a sudden change in the confirmed cases, which seems more effective than the direct estimation of the number of new cases.

Biswas *et al*. fit the cumulative data of COVID-19 to an empirical SIR model combined with a Euclidean network.^14^ Evaluating the volume of confirmed cases versus the geographical distance to the epicentre for both China and Italy, they discover an incredibly robust spatial dependency in the outbreak of COVID-19 showing an approximate power-law variation with an exponent of 1.85. It is also demonstrated that the SIR model, combined with the Euclidean network, can develop better reliability based on the data in China.

### Basic reproduction number

The basic reproduction number *R*_0_ is an essential indicator in epidemiology to represent whether an infectious disease develops a pandemic. Under the SIR model, *R*_0_ represents the average number of secondary cases transmitted by a single infected individual that is placed into a fully susceptible population.^15^ In mathematical express *R*_0_ is defined as the maximum eigenvalue of the next-generation matrix *G*, where the *ij* element is the expected number of type *i* cases as a result of infectious individuals of type *j*. More specifically, if *R*_0_ is higher than one, the epidemic spreads rapidly. In contrast, if *R*_0_ is less than one, the epidemic propagates slowly and vanishes before everyone gets infected.

Wu *et al*. formulate a refined SIR model by incorporating domestic transportation data to predict the size of the epidemic in Wuhan.^16^ The dataset used here is based on monthly flight bookings and human mobility across more than 300 cities in China. The estimation of *R*_0_ is approximately 2.68 (2.47–2.86, 95% CI) based on the data in Wuhan. One limitation is that their assumption is based on the fact that the COVID-19 cannot change travel behaviour, and all infectious individuals are ultimately detectable with symptoms.

Riou and Althaus estimate *R*_0_ using negative-binomial offspring distribution with mean *R*_0_ and dispersion parameter *k* from a simulation, where the *k* quantifies the variability in the number of secondary cases.^17^ The generation time interval *D* is presumed to follow a gamma distribution. The simulation runs with various combinations of parameters, which estimates that *R*_0_ is 2.2 (1.4–3.8, 90% CI) assuming the number of infected cases increased from 1000 to 9700 by 18 January 2020. One constraint of this approach is that it is based on a relatively small number of data points. However can be extended to a wide selection of parameters by running more simulations.

Liu *et al*. estimate *R*_0_ by applying a prior Gamma distribution to fit the generation time, assuming that the cases are in an exponential rate of growth.^18^ They use Poisson regression to determine the exponential growth rate, and estimate time-varying instantaneous reproduction number *R*_0_(*t*), which signifies the *R*_0_ at time *t* if conditions stay constant after time *t*.^18^ The *R_t_* is expected similarly as *R*_0_ but uses the past ten-day moving window. Chinese national estimation of *R*_0_(*t*) is 4.5 (4.4–4.6, 95% CI). *R_t_* increases from 6.9 (5.5–8.4, 95% CI) to peak 8.8 (8.3–9.4, 95% CI) by 16 January 2020, then continuously decreases to 1.59 (1.57–1.61, 95% CI) by 6 February 2020. The drawback of this research is that the incidence date is predicted by a generalized additive model, which may result in misclassification bias.

Read, *et al*. calibrate the model taking into account of air traveller with an assumption that daily growth of confirmed cases follows a Poisson distribution.^19^ The confirmed cases reported from 1 January 2020 to 22 January 2020 is chosen to fit the model. Maximum likelihood estimation (MLE) is employed for parameter evaluation. The value of *R*_0_ is estimate to be 3.11 (2.39–4.13, 95% CI). The major limitation of the model is that it only includes a spatial component of the model according to airline travelling, but does not include other transportations such as rail and road. As a result, it probably underestimates local connectivity and the connectivity of Wuhan to other locations.

Hong and Li extend the standard SIR model by introducing time-independent variables to capture the dynamics of transmission and removal rates.^20^ The literature employs a Poisson model with a polynomial approximation to estimate an instantaneous basic reproduction number *R*_0_(*t*), which presents a real-time approximation of *R*_0_ in the epidemic. They also validate the model by analysing the data from various severely affected countries, which demonstrates that the dynamics of the outbreak can be estimated. Although the time-dependent *R*_0_(*t*) is effective to assess the effectiveness of prevention measures in real time, the main restriction is that the model does not consider the impact of asymptomatic infections, which possibly underestimates the *R*_0_(*t*).

### Asymptomatic transmission

In the outbreak of COVID-19, the infected individuals may have a broad spectrum of symptoms. However, it is also possible that vast numbers of people have no symptoms, so-called asymptomatic cases, due to the unawareness of infections or the limited capacity of testing. If an infected individual has no symptoms, it is complicated to detect the outbreak considering that they can be spreading the virus without even knowing they are doing so. Therefore, asymptomatic transmission is the most challenging part in controlling the ongoing COVID-19 epidemic.

The SEIR model is another extension to the basic SIR model in order to consider asymptomatic transmission. SEIR provides a practical approach to model a disease in a considerable incubation period when the exposed person is not yet infectious.

**Figure 4.**
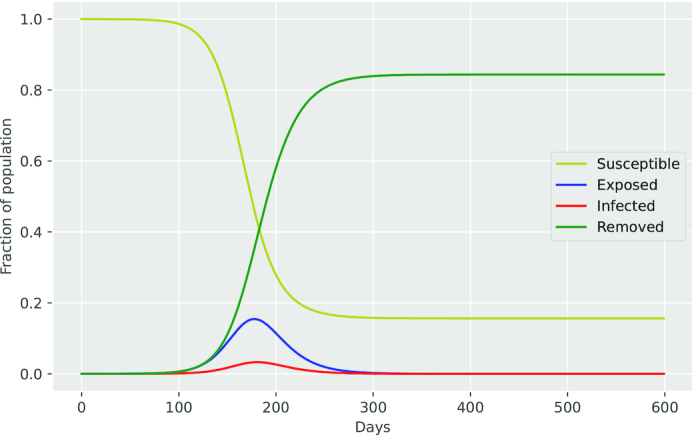
SEIR model.

As shown in Fig. 4 the SEIR model provides a new class named Exposed (*E*), which includes the population in the incubation period. The equations can be formulated as follows.

\begin{eqnarray*} \frac{d S}{d t} &=& -\beta S I+ (\lambda -\mu ) S \\ \frac{d E}{d t} &=& \beta S I- (\mu +k) E \\ \frac{d I}{d t} &=& k E- (\gamma +\mu ) I \\ \frac{d R}{d t} &=& \gamma I-\mu R \end{eqnarray*}

where β indicates the effective contact rate; λ implies the birth rate of susceptibility; *μ* represents the mortality rate; and*k* signifies the progression rate from the (latent) exposed to the infected; and *γ* is the the removal rate.

Ferretti *et al*. specify four categories in the transmission of the virus: symptomatic, asymptomatic, pre-symptomatic, and environmental transmission.^21^*R*_0_ is the summation of the four parts of the basic reproduction number. Using Bayesian methods and Maximum Likelihood Estimation (MLE), the approximation of *R*_0_ is around 2.0 (1.7–2.5, 95% CI), with a fraction of the four categories: pre-symptomatic 0.47 (0.11–0.58 CI), symptomatic 0.38 (0.09–0.49 CI), environmental 0.1 (0.02–0.56 CI), and asymptomatic 0.06 (0–0.57 CI). The results suggest a high proportion of pre-symptomatic transmissions. It is also noticeable that the estimation of *R*_0_ in this literature is slightly smaller in comparison to the other results, e.g. Refs. 16–19. This is due to the assumption that COVID-19 gives shorter generation times. Therefore a smaller portion of infections can be obstructed by the suppression (*R*_0_ < 1). This does not mean suppression is quicker to achieve. In contrast, the transmissions develop in a faster manner, and a larger portion gets infected prior to the symptoms.

Li, *et al*. modify the SEIR model to identify the undetected cases taking account of the mobility between 375 Chinese cities.^22^ A mathematical model is implemented to examine the transmission dynamics during the early phase of the outbreak through simulating observations between 10 January 2020 and 23 January 2020. The model parameters are inferred by the use of an iterated filter-ensemble adjustment Kalman filter (IF-EAKF) framework.^23^ The estimation of *R*_0_ is 2.38 (2.04–2.77, 95% CI) at the beginning of the epidemic. Additionally, it is also predicted that only 14% (10–18%, 95% CI) of total infections in China are reported. The range of *R*_0_ for COVID-19 is between 1.4 and 4.6, which is higher than the *R*_0_ of Middle East Respiratory Syndrome (MERS) (*R*_0_: 0.3–0.8) and close to Severe Acute Respiratory Syndrome (SARS) (*R*_0_: 2–5).^6^

Jenny *et al*. extend the SIR model with the undetected cases, which can be transformed into detected cases by testing.^24^ Model parameters are estimated using the confirmed cases of COVID-19 outside China from 22 January 2020 to 12 March 2020. The results clearly show that mass-testing and follow-up isolation of confirmed cases are the effective mitigation strategy.^24^ The model can be further improved by taking more reliable data, and considering diverse age groups and the latency period.

Yuan *et al*. build a standard framework to forecast a turning period during the outbreak of an epidemic.^25^ They extend the classical SEIR modal to an individual SEIR and use observed daily cases in Wuhan as the input to the model. The development of dynamics of the epidemic in the subsequent days can be estimated, which shows that the model can be used to forecast the turning period, i.e. one week following 14 February 2020, for the outbreak in Wuhan. This prediction is recognized as prompt and precise. However, this model is still based on a deterministic one and can be improved to use stochastic models, such as the Markov chain.

### Herd immunity

Herd immunity is a fundamental concept in epidemic theory regarding the population-level effect of individual immunity to prevent transmission of infections.^26^ A population is considered to have herd immunity for a disease if a sufficiently large proportion of the population possess immunity against the virus, so the chance of active contact between infected and susceptible people is minimized.^27^ Herd immunity is influenced by numerous aspects such as virus dynamics and transmission modes, along with the individuals in the population who acquire immunity.^28^

Mathematically, if the population is homogeneously mixing, herd immunity can be achieved if a large enough uniformly distributed population is immune.^26^ In order to prevent the outbreak of an infectious virus, a sufficient amount of people must be immune to assure the susceptible fraction is small enough, which means that the average infective number is smaller than one. This may occur in two ways: (i) natural immunity—lots of individuals contract the virus in time and develop an immune response; and (ii) vaccination—a significant number of people are vaccinated against the disease. For several infections, herd immunity may go into effect when 40% of the people in a population become immune to the disease. Nevertheless, in many cases, 80%–95% of the population needs to be immune to stop its spread.^27^

Lourenco *et al*. build the SIR models to investigate the sensitivity to the specific percentage of the population at risk of severe disease and death.^29^ They calibrate the models using the cumulative confirmed deaths of COVID-19 in the UK and Italy under the assumption that such deaths are well-recorded and occur only in a vulnerable fraction of the population. Various models are used based on prior estimations of essential epidemiological parameters such as the *R*_0_, CFR, transmittable period, and the period from infection to death, and so on. The models are formulated with *R*_0_ = 2.25 and *R*_0_ = 2.75 separately. It is estimated that by 19 March 2020 approximately 36% of the UK population might have been exposed to COVID-19 if *R*_0_ = 2.25 and 40% if *R*_0_ = 2.75.^29^ An identical experiment is performed with assumptions that *R*_0_ is 2.25 and the proportion of the population susceptible to severe disease is 0.1%. The preliminary result reveals that 68% of the UK population could have been infected by 19 March 2020. Sir Patrick Vallance, Chief scientific adviser to the UK Government, claims that nearly 60% population is desired in order to achieve herb immunity for COVID-19. If the modelling is legitimate, the result may suggest that the UK could have already developed herd immunity. However, this research is based on a theoretical simulation with an early-stage dataset, which makes the model relatively sensitive to a multitude of simplified assumptions. For example, it assumes the population is well-mixed, which implies the model maybe overestimate the transmission rate and consequently the infected proportion. If it assumes the proportion of the population at risk is 1%, the infected proportion is dramatically reduced to 36% and 40%.

### Effect of intervention measures

Preventative measures, with intention to lower transmission rates in the population and thus reduce the infection of the virus, are essential for an infectious outbreak when a vaccine is not available. The prevention and containment strategies can be broadly considered in two categories: (i) pharmaceutical interventions, including antivirals and vaccines; and (ii) non-pharmaceutical interventions (NPIs), including case isolation, household quarantine, shop, school or workplace closure, restrictions on travel, and so on.^30^ Some governments impose a series of tight NPIs to slow down the spread of the virus, while the other nations promise to implement more severe control measures if necessary in the future. Mathematical models are effective tools to investigate the sophisticated situation with intervention strategies and estimate the potential benefits and costs of different strategies. Numerous studies have been carried out to facilitate the policymaker to select different interventions.

Tian *et al*. apply regression models to examine the transmission control measures in China.^1^ The prevention measures of COVID-19 are investigated using specific data from China, including case studies, human activities, and intervention measures. It is found that the measures highly likely postpone the outbreak of the epidemic and lead to a decline in terms of the number of confirmed cases during the 50 days of the lockdown period in China. It is also shown that the shutdown of Wuhan city has delayed outbreak in other cities by at least 2.91 days (2.54–3.29: 95% CI). Cities that implemented control measures reportedly demonstrate fewer cases in the first week of their outbreaks (13.0; 7.1–18.8) in contrast to the cities that started control later (20.6; 14.5–26.8). This study is based on the data in China from 31 December 2019 to 19 February 2020. So it is still an early result and does not necessarily present the full impact of all aspects of NPIs in a large area.

For control measures that are already taken, Kraemer *et al*. employ the dataset, including confirmed cases and travel history, to evaluate the impact of control measures.^31^ They construct three models, namely Poisson Generalized Linear Model (GLM), negative binomial GLM, and log-linear regression with four parameters, i.e. the number of cases, an indicator of test availability of real-time quantitative polymerase chain reaction (RT-qPCR) , estimated mobility, an indicator of date before or after 26 January 2020.^31^ They perform model selection to estimate daily confirmed cases and discover that models incorporating the mobility data into regression models can predict the volume of cases with more precise results. It is concluded that the travel restrictions enforced in China significantly suppressed the outbreak of COVID-19.

Prem and Cook use geographical contact information of various age groups in Wuhan to investigate how shifts in population groups influence the progress of the outbreak in Wuhan.^32^ They simulate the outbreak in Wuhan using SEIR for 16 age groups and refine the model with location-based distancing measures such as school closures. The results reveal that the uncertainty of *R*_0_ has a substantial effect on the peak time of the outbreak and the ultimate scope of the epidemic and social distancing measures are most effective when the people return to work. This research is unable to definitively identify the effect of each NPIs due to a deficiency of data.

For potential interventions that could be effective in the future, Ferguson *et al*. evaluate the potential impact of public health measures on the transmission rate of the virus. They consider two fundamental categories of strategies: (i) mitigation focusing on delaying the epidemic spread and (ii) suppression aiming at slow epidemic growth. They assess the risk of sustained transmission by simulating two models on data from the UK and the US.^33^ They use a stochastic, spatially structured individual-based simulation proposed in ref.^34^ The model is determined by the population in a geographic region that can be simulated by Poisson distributed with parameters driven by the density of population. It also assumes that transmission occurs in three sources: household, schools and workplaces, and community. Community transmission depends explicitly on distance, i.e. the probability that individual *i* infects individual *k* is weighted by a kernel function *f*(*d_i, k_*), where *d_i, k_* is the distance between individuals *i* and *k*. For any time step Δ*T*, a susceptible individual *i* has probability 1 − exp(−λ_*i*_Δ*T*) of being infected, where λ_*i*_ is the instantaneous infection risk for individual *i*.^30^ Markov chain Monte Carlo (MCMC) and Maximum Likelihood Estimation (MLE) are applied to infer the posterior distribution of model parameters including latent period, infectiousness over time and transmission coefficients. They test five NPIs in the three sources of transmission and conclude that the overall performance of a single intervention is perhaps limited, in need of a combination of intervention measures to be reinforced to have a substantial impact on transmission. It is uncovered that the peak healthcare demand can be reduced by two-thirds, and the deaths can be reduced by half as a result of mitigation policies incorporating home isolation, home quarantine, and social distancing. It is also proven that suppression also demands a mixture of social distancing, home isolation, and quarantine in both the UK and the US.

## Conclusions

We have reviewed a range of mathematical models for the outbreak of COVID-19, which can be employed to estimate the epidemiological trend including severity (Case Fatality Ratio), basic reproduction number, and herd immunity as well as the potential effects of interventions on COVID-19. The mathematical modelling for an ongoing outbreak is still a challenging task at this stage of the epidemic. We find that most existing epidemiological models of COVID-19 are typically based on epidemic-dynamic models rather than the statistical models or machine learning. There is still considerable uncertainty to estimate the epidemiological characteristics due to the novel nature of COVID-19 and the early stage of the outbreak. Certain methods present some inherent limitations as a result of insufficient data and limited data sources. Additional research is urgently necessary to fulfil such research gaps.

We believe that as the epidemic reaches to the end and more data can be collected, epidemiological models can be improved to present a real reflection of the full picture. It is noticeable that studies relating to either current or future prevention measures only consider the effect on minimizing the spread of the pathogen but neglect the economic costs of interventions that are also crucial for policymaking. Research on prevention measures considering the constraint of economic costs is deemed as the future works of this research.
